# Qi Dan Li Xin pill improves chronic heart failure by regulating mTOR/p70S6k-mediated autophagy and inhibiting apoptosis

**DOI:** 10.1038/s41598-020-63090-9

**Published:** 2020-04-08

**Authors:** Binhao Shi, Yuting Huang, Jingyu Ni, Jingrui Chen, Jing Wei, Hui Gao, Lan Li, Zhengcan Zhou, Yili Wang, Yunsheng Xu, Zongpei Xu, Jingyuan Mao, Guanwei Fan

**Affiliations:** 1 First Teaching Hospital of Tianjin University of Traditional Chinese Medicine, Tianjin Key Laboratory of Translational Research of TCM Prescription and Syndrome, Tianjin, China; 2 LongHua Hospital Shanghai University of Traditional Chinese Medicine, Shanghai, China; 3 Tianjin State Key Laboratory of Modern Chinese Medicine, Tianjin University of Traditional Chinese Medicine, Tianjin, China

**Keywords:** Heart failure, Molecularly targeted therapy

## Abstract

Myocardial remodeling represents a key factor in chronic heart failure (CHF) development, and is characterized by chronic death of cardiomyocytes. Cardiac function changes may be attributed to inflammation, apoptosis and autophagy. This study assessed the effects of Qi Dan Li Xin Pill (QD) on heart function, inflammatory factors, autophagy and apoptosis in cardiac remodeling in CHF rats upon myocardial infarction (MI) induction. Male SD rats underwent a sham procedure or left anterior descending coronary artery (LADCA) ligation, causing MI. Twenty-eight days after modeling, the animals were treated daily with QD, valsartan and saline for 4 weeks. Echocardiography after 4 weeks of drug intervention revealed substantially improved left ventricular remodeling and cardiac function following QD treatment. As demonstrated by decreased IL-1β, IL-6 and TNF-α amounts, this treatment also inhibited the apoptotic process and protected the viability of the myocardium. These outcomes may be attributed to enhanced autophagy in cardiomyocytes, which further reduced pro-inflammatory and pro apoptotic effects. This process may be achieved by QD regulation of the mTOR/P70S6K signaling pathway, suggesting that the traditional Chinese medicine Qi Dan Li Xin pill is effective in heart protective treatment, and is worth further investigation.

## Introduction

Heart Failure (HF) represents a complex ailment that mainly results from cardiomyopathy, abnormal cardiac load and arrythmias, including acute heart failure and CHF. In CHF, the cases present clinical manifestations such as breathlessness at rest or during exercise, fatigue, tachycardia, tachypnoea, peripheral edema, structural or functional abnormalities of the heart, and others. CHF constitutes a severe phase and the terminal stage of multiple cardiac ailments, and is considered a major cardiovascular disease in China^1,2^. Early and/or targeted treatment could delay the progression of heart failure.

Irreversible cardiomyocyte death is the main cause of CHF, structurally and functionally damaging the heart. Two cell death types are known in CHF, including necrosis and apoptosis^3^. Specifically, myocardial infarction (MI)-induced chronic heart failure features substantial systemic and local inflammatory reactions^4^. Damage-associated molecular patterns (DAMPs) release a large number of inflammatory factors that continue to damage cardiac myocytes and might induce innate immunity, exacerbating cardiomyocyte injury^5,6^. Myocardial cell necrosis and apoptosis are intertwined after MI. In addition, myocardial apoptosis is well-documented in the progression of chronic heart failure^7^.

Autophagy is an evolutionarily conserved mechanism, which mainly uses lysosomes to degrade excess, aging or damaged cytoplasmic materials. It represents an important physiological activity to maintain cell homeostasis, growth, differentiation, development and environmental adaptability. Autophagy occurs at baseline level under non-pathological conditions, but is triggered by multiple microenvironmental inducers such as biological, hormonal, nutritional, metabolic and physicochemical cues. In most cases, autophagic reactions help cells adapt to stressors, promoting cell viability^8^. The optimal autophagic level is critical to cardiac function maintenance. It was demonstrated autophagy occurring in cardiac myocytes under ischemia can supply needed energy for the survival of cells via removal of disabled organelles or aging proteins^9,10^. Furthermore, autophagy upregulation may be a mechanism to alleviate further myocardial injury and cardiac insufficiency after MI^11,12^. Moreover, mTOR signaling is one of the most important pathways tightly associated with cell apoptosis and autophagy; it plays important roles in cellular biological processes, including cell apoptosis, transcription, translation, metabolism, angiogenesis and cell cycle regulation)^13^, making it a suitable pathway to investigate.

Qi Dan Li Xin Pill (QD), a patent of First Teaching Hospital of Tianjin University of Traditional Chinese Medicine (Patent No. ZL201110413471.1), is an achievement from the process of long-term treatment of heart failure. Previous findings indicated that QD could effectively relieve clinical symptoms and improve heart function in patients with CHF^14^. Here, QD’s effects on autophagy were assessed *in vivo*. In addition, QD-associated regulation of mTOR signaling was explored. Our findings provide novel insights into the mechanisms underpinning QD’s effects in CHF treatment.

## Results

### Echocardiographic findings

**Figure 1:**
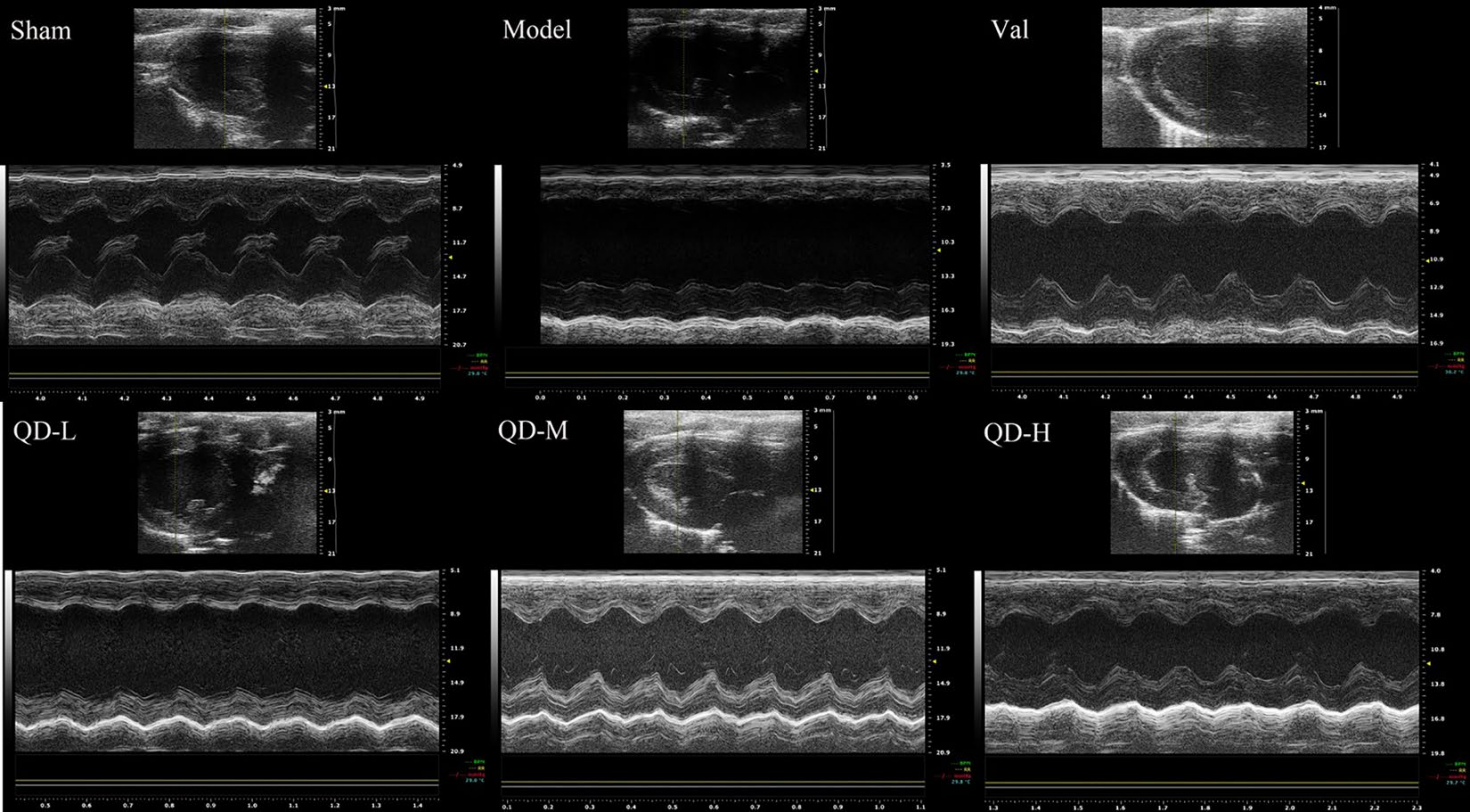
(Fan). Representative echocardiographic images (m-mode) in various groups. Compared with sham animals, the model group showed overtly damaged left ventricular diastolic and systolic functions, significantly weakened cardiac compliance, strengthened myocardial hardness, and obvious ventricular remodeling. However, QD significantly delayed ventricular remodeling, and progressive deterioration of heart function was effectively inhibited.

**Figure 2:**
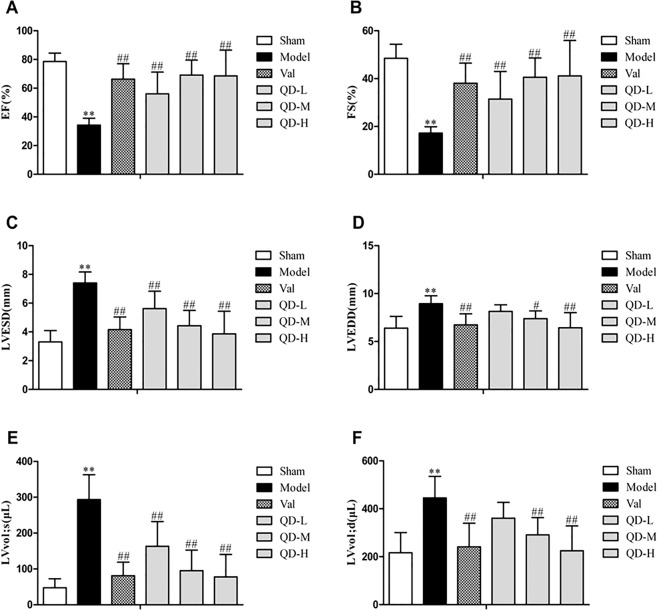
(Fan). Effects of QD on rat heart function. (**A**) LVEF, LV ejection fraction; LVFS, (**B**) LVESD, LV fractional; (**C**) LV end-systolic diameter; (**D**) LVEED, LV end-diastolic diameter; (**E**) LVvol;s, LV end-systolic volume; (**F**) LVvol;d, LV end-diastolic volume. n = 6/group. Data are mean ± s.d.; **P < 0.01 versus sham group; ^#^P < 0.05 and ^##^P < 0.01 versus model group.

All animals had normal cardiac function before surgery. In comparison with sham animals, model rats showed remarkable changes in echocardiographic data, but the systolic function of the heart was significantly enhanced by intragastric administration (Figs. 1, 2). Echocardiography revealed that EF, FS, LVESD, LVEDD, and LV vol were improved after drug intervention. Compared with the coronary ligature group, the treatment groups showed marked differences in EF and FS, which were overtly raised after QD and valsartan administration for 28 days intragastrically (P < 0.01) (Fig. 2A,B). On one hand, regional wall motion anomalies were relieved in the treatment groups, including LVESD, with a significant difference versus the coronary ligature group (P < 0.01) (Fig. 2C). On the other hand, both QD and valsartan significantly reduced cardiac load in rats with CHF, as observed with left ventricular end systolic volume (P < 0.01) (Fig. 2E). These findings demonstrated that QD had similar or even better effects compared with valsartan.

### Hemodynamics

**Figure 3:**
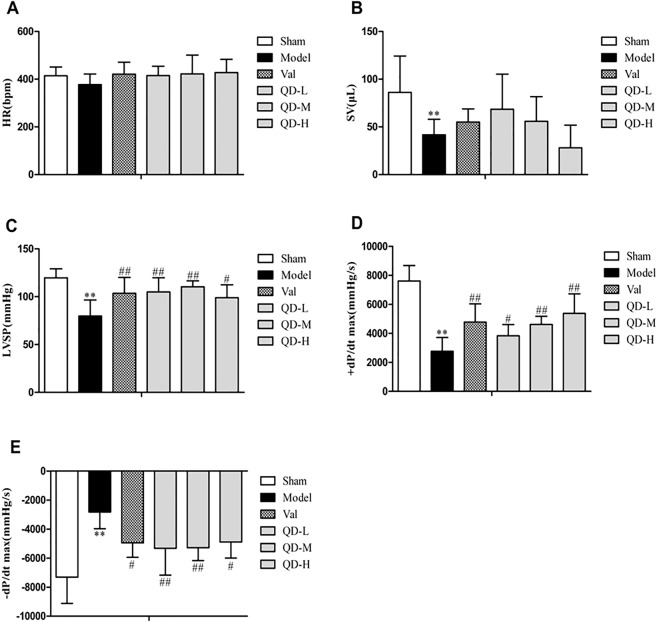
(Fan). Quantitative evaluation of hemodynamic properties of the heart. Heart rate (HR) (**A**), stroke volume (SV) (**B**), left ventricular systolic pressure (LVSP) (**C**), left ventricular maximum descent velocity (−dp/dt max) (**D**), and left ventricular maximum upstroke velocity (+dp/dt max) (**E**) were assessed. n = 6/group. Data are mean ± s.d. **P < 0.01 versus sham group; ^#^P < 0.05 and ^##^P < 0.01 versus model group.

We used hemodynamics to further confirm the improvement of cardiac function by QD. In comparison with untreated model animals, the QD-L, QD-M and valsartan groups showed markedly increased left ventricular systolic pressure (LVSP) (P < 0.01) (Fig. 3). In addition, QD-M, QD-H and valsartan significantly enhanced the maximum rate of rise of ventricular pressure (+dp/dt max) (P < 0.01). Meanwhile, QD-L and QD-M could remarkably improve the maximum rate of reduction of ventricular pressure (-dp/dt max) (P < 0.01). However, various treatment groups had no significant differences in heart rate (HR) and stroke volume (SV) in comparison with uncreated model animals. In comparison with other treatment groups, the QD-M group showed progressively improved cardiac function in rats with CHF.

### QD inhibits inflammation in chronic heart failure

**Figure 4:**
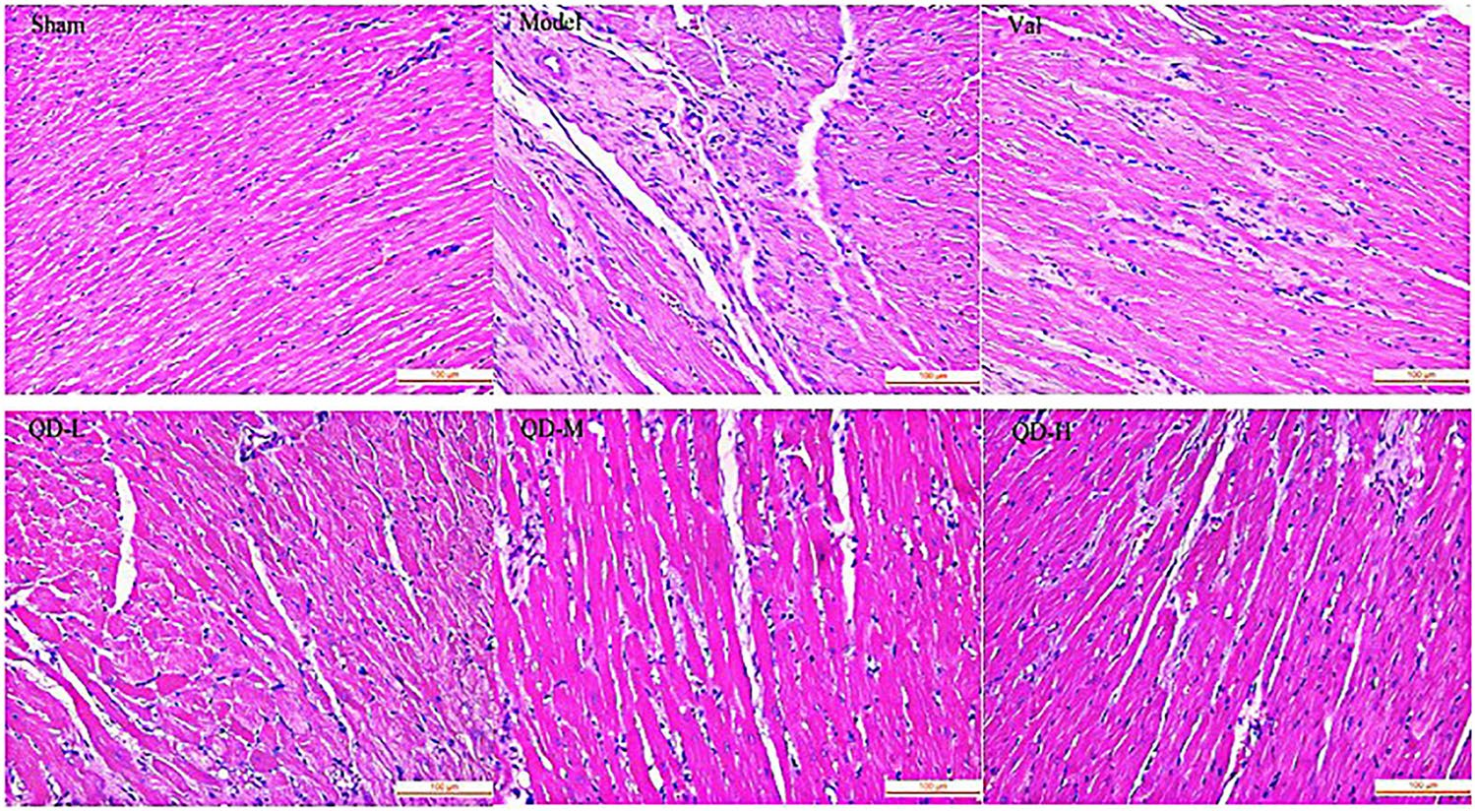
(Fan). Histopathological assessment of the left ventricle at 28 days post-CHF induction. In rats with myocardial infarction, more fibroblasts at the edge of the infarcted area showed hyperplasia, more live myocardial cells displayed hypertrophy, and less mononuclear cell infiltration was observed compared with the sham group. In the QD groups, fibroblast amounts were obviously reduced at the edge of the infarcted area, with decreased inflammatory cell infiltration, in comparison with the model group (magnification, 400×).

By H&E staining of heart slices, infiltration of inflammatory cells, hypertrophy of cardiac myocytes, edema and loosening, fibroblast proliferation and fibrous scar formation in the marginal zone were observed in model rats with CHF. In comparison with model animals, the QD and valsartan groups exhibited improved inflammation, myocardial cell morphology and scar repair to varying degrees, and maintained the integrity of myocardial cells (Fig. 4).

**Figure 5:**
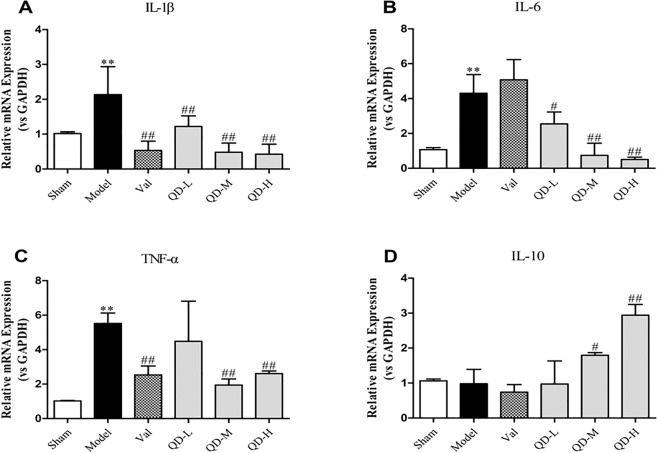
(Fan). Cardiac IL-1β, IL-6, TNF-α and IL-10 mRNA amounts in rats 28 days post-CHF induction. Gene expression was examined by qRT-PCR. n = 3 per group. GAPDH was employed for normalization. Data are mean ± s.d. **P < 0.01 versus sham group; ^#^P < 0.05, ^##^P < 0.01 versus model group.

In order to confirm the anti-inflammatory effects of QD, qRT-PCR was performed for assessing the mRNA amounts of myocardial inflammatory factors **(**Fig. 5**)**. The results showed that IL-1β, IL-6 and TNF-α mRNAs were highly expressed in untreated model animals with CHF (P < 0.01), but were lowered in the QD and valsartan groups. Notably, IL-1β (P < 0.01, Fig. 5A), IL-6 and TNF-α mRNA amounts were significantly decreased in both QD-M and QD-H groups (P < 0.01, Fig. 5B,C). In addition, QD reduced the secretion of inflammatory factors to a higher degree compared with valsartan. Furthermore, QD promoted IL-10 secretion **(**Fig. 5D**)**.

### QD reduces cardiomyocyte apoptosis in chronic heart failure

Caspase 3 and Bax are key molecules affecting myocardial apoptosis in CHF after MI. High expression levels of caspase 3 and Bax indicate active cardiomyocyte apoptosis, while increased expression of Bcl-2 inhibits cardiomyocyte apoptosis; therefore, the Bcl-2/Bax ratio could reflect the degree of apoptosis in cardiomyocytes.

**Figure 6:**
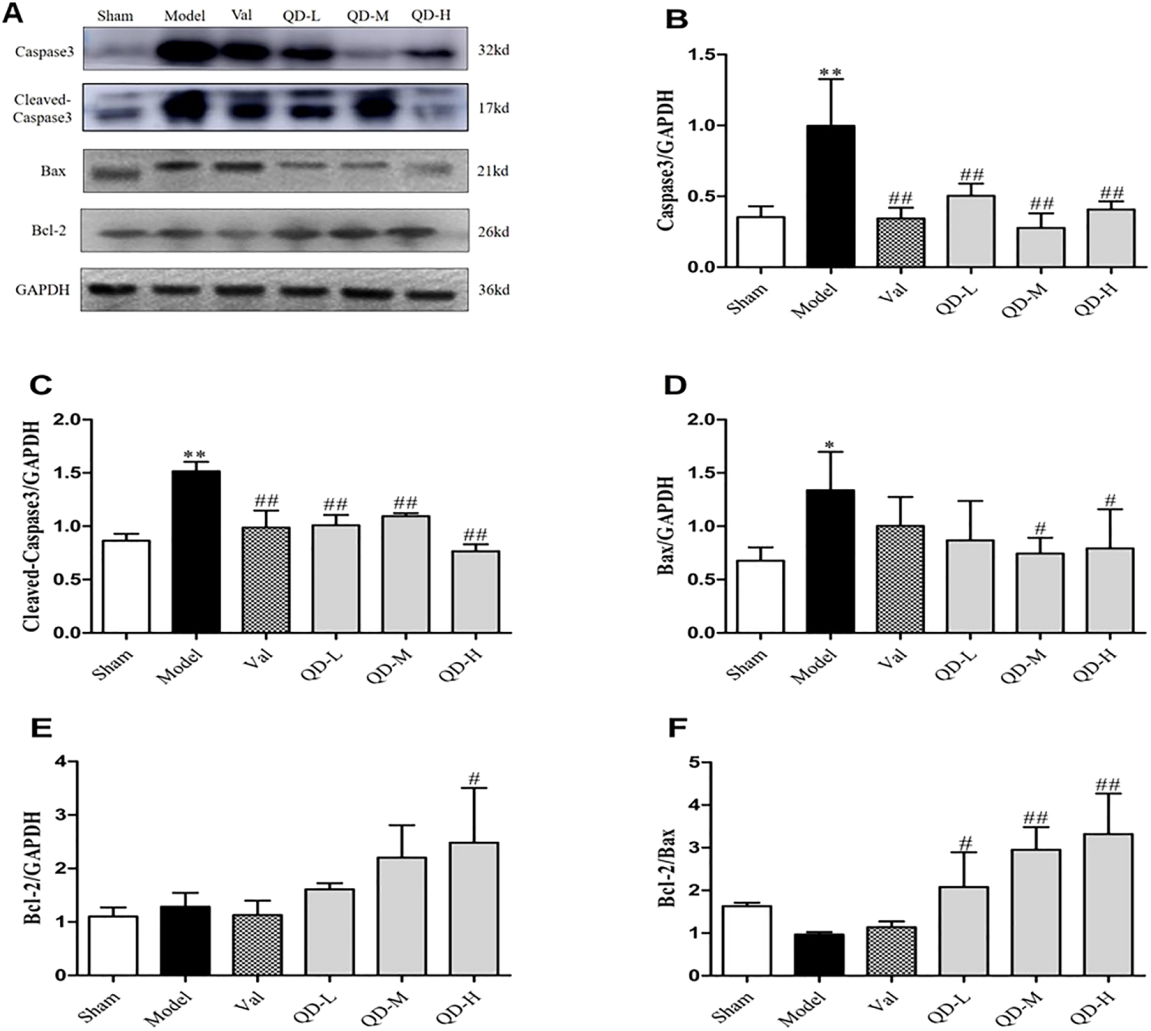
(Fan). Protein amounts of apoptosis-associated molecules. (**A**) Representative immunoblot bands of various proteins. (**B–F**) Semi-quantitative evaluation of caspase3, cleaved-caspase3, Bax, Bcl-2 and Bcl-2/Bax in various groups. Data are mean ± s.d. *P < 0.05, **P < 0.01 versus sham group; ^#^P < 0.05, ^##^P < 0.01 versus model group (n = 3 per group).

To verify QD’s protective effects on the heart in CHF, rat hearts after 4 weeks of treatment were extracted and subjected to Western blotting. As shown in Fig. 6, caspase 3 and Bax proteins were upregulated in CHF hearts (P < 0.01, P < 0.05), but markedly downregulated by QD and valsartan, while increased levels of the anti-apoptotic molecule Bcl-2 were observed. Furthermore, QD-M and QD-H showed pronounced protective effects on myocardial cells, markedly reducing the level of myocardial apoptosis (P < 0.01, P < 0.05).

### QD may delay heart failure progression by regulating the mTOR/p70S6k signaling pathway

**Figure 7:**
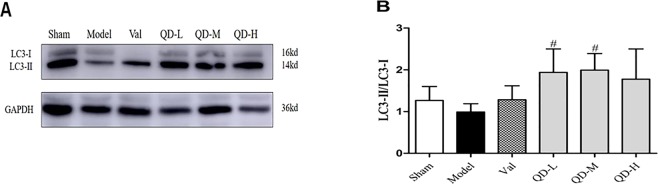
(Fan). Protein amounts of autophagy-associated molecules. (**A**) Representative immunoblot bands of various proteins. (**B**) Semi-quantitative evaluation of LC3-II/LC3-I in various groups. Data are mean ± s.d. *P < 0.05, **P < 0.01 versus sham group; ^#^P < 0.05, ^##^P < 0.01 versus model group (n = 3 per group).

Microtubule-associated protein 1 A/1B light chain 3 (LC3) represents an important molecule controlling autophagy; LC3′s cytosolic form (LC3-I) interacts with phosphatidylethanolamine to generate LC3-II, which is recruited to autophagosome membranes^15^. LC3-II turnover in lysosomes is associated with starvation-related autophagy. Western blot showed that LC-II protein amounts were markedly elevated in the drug groups **(**Fig. 7A**)**. Moreover, the LC3II/LC3I ratio was starkly increased than that of untreated model animals **(**Fig. 7B**)**, particularly after treatment with QD-L and QD-M groups (P < 0.05). This finding indicated that QD could promote or maintain autophagic activity in the infarcted area.

**Figure 8:**
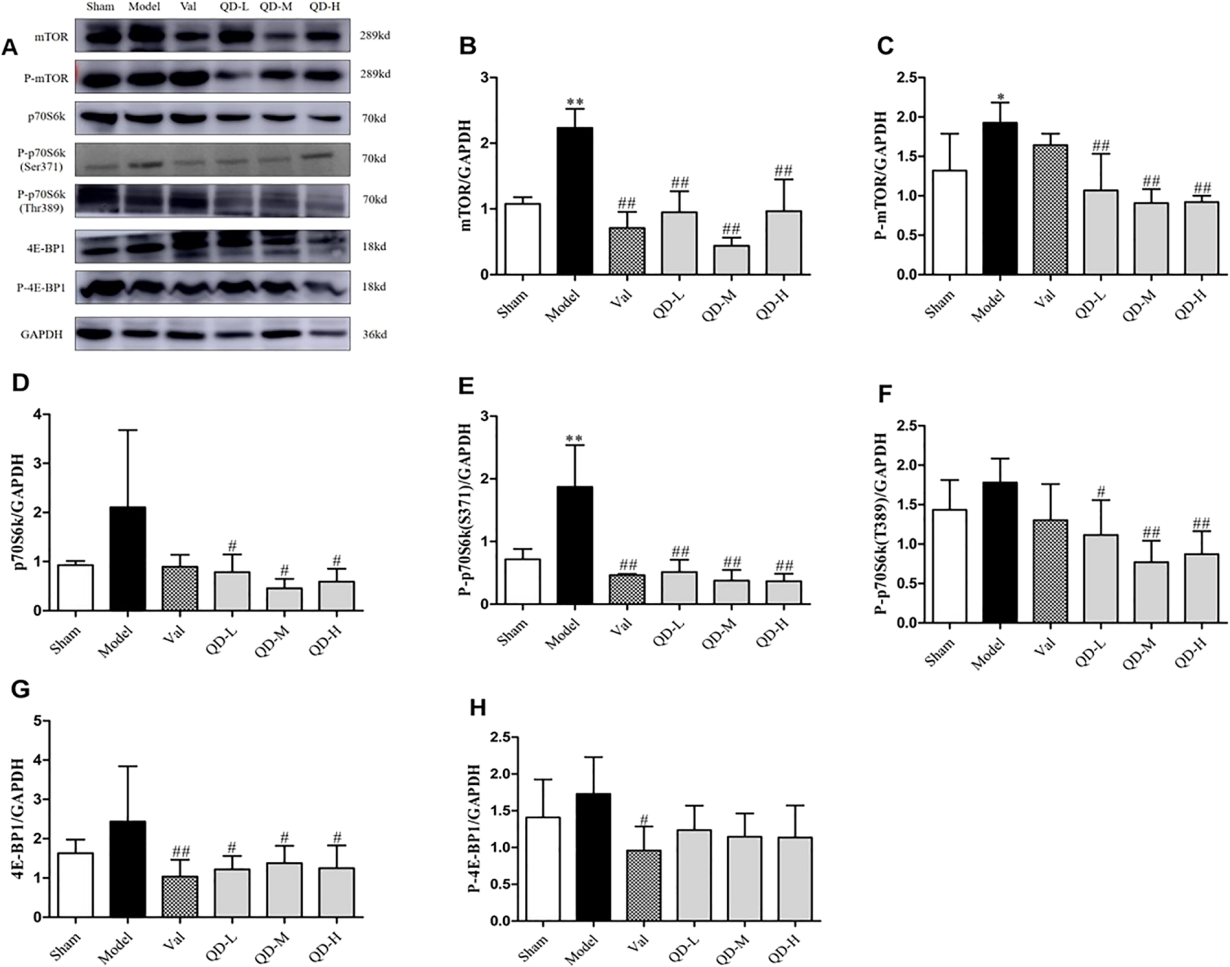
(Fan). Protein amounts of mTOR pathway effectors. (**A**) Representative immunoblot bands of various proteins. (**B–H**) Semi-quantitative assessment of mTOR, P-mTOR, p70S6k, P-p70S6k (S371), P-p70S6k (T389), 4E-BP1, P-4E-BP1. Data are mean ± s.d. *P < 0.05, **P < 0.01 versus sham group; ^#^P < 0.05, ^##^P < 0.01 versus model group (n = 3, each group).

Evidence shows that mTOR has a significant function in promoting cardiac diseases, including cardiomyopathy, myocardial ischemic injury and myocardial fibrosis^13^. Furthermore, overexpression of the mTOR complex promotes the progression of heart failure^8^. In comparison with sham animals, the model group of chronic heart failure rats showed significantly increased mTOR and phosphorylated mTOR (P-mTOR) amounts **(**Fig. 8B,C; P < 0.01, P < 0.05), which confirmed that mTOR might have an important function in CHF. On the other hand, QD significantly inhibited mTOR phosphorylation, suggesting that mTOR is one of the targets of QD. Next, we detected the phosphorylation of mTOR substrates, including 4E-BP1, S6K, and S6. The results showed that the trends of p70S6k and P-p70S6k expression were basically consistent with mTOR amounts. Compared with the model group, QD treatment resulted in remarkably reduced p70S6k and P-p70S6k (site 371/389) amounts (P < 0.05, P < 0.01). However, 4E-BP1 phosphorylation was not significantly altered by QD.

## Discussion

Heart failure is a chronic, spontaneous progressive disease. Due to cardiomyopathy, abnormal cardiac load and arrhythmia are its main causes; early or targeted treatment should delay heart failure progression. According to traditional Chinese medicine (TCM), the causes of chronic heart failure mainly include Qi deficiency of vital energy, blood stasis and phlegm retention. As a result, therapeutic principles and methods for CHF are termed “Yiqi Huoxue and Lishui” (Tonifying Qi, Activate blood, and removing water retention)^16^. QD, one of the representative prescriptions, is mainly composed of *Astragalus membranaceus*, *Salvia miltiorrhiza*, lepidium seed, poria cocos and others. According to the principles of Traditional Chinese Medicine about Jun (Emperior) Chen (Minister) Zuo (Adjuvant) Shi (Guiding), we found that QD has good effects in alleviating CHF in clinical practice, e.g., improving the quality of life, increasing 6-miniute-walk distance and ejection fraction^17^. Multiple studies have assessed the efficacy of drugs that make up QD in the treatment of cardiovascular diseases, which do not need further investigation. The current work confirmed and expanded our previously reported findings, demonstrating that QD could regulate cardiac remodeling and homeostasis.

Here, we showed that CHF after MI is accompanied by myocardial inflammation, apoptosis and ventricular remodeling. After MI, inflammatory reactions are triggered by DAMPs, activating macrophages to coordinate inflammation and tissue repair; in addition, macrophages regulate autophagy to exert innate immune function^5,18^. Activating subsequent inflammatory/reparative pathways, the highly inflammatory factors trigger apoptosis in cardiomyocytes, cardiac hypertrophy and stiffness, differentiation of myofibroblasts, collagen accumulation, endothelial dysfunction, and endothelial-to-mesenchymal transition, and ensuing ventricular remodeling and impaired left ventricular function. Continuous inflammatory stimulation promotes apoptosis in cardiac myocytes; even though a compensatory process may occur, the number of functional cardiac myocytes may still decrease. Indeed, many reports suggest that low-grade chronic inflammation might represent an essential contributor to the maintenance or clinical deterioration of patients with established CHF, with inflammatory factors (e.g., IL-1β, IL-6, and TNF-α) playing critical roles^19–21^. As shown above, QD reduced inflammatory cytokine (IL-1ß, IL-6 and TNF-α) levels in CHF rats, while increasing IL-10 amounts.

A report showed that apoptosis participates in macrophage dispersal during development, with chronic high amounts of uncleared apoptotic cells and acute apoptosis induction impairing wound responses in experimental studies, without requiring phagocytosis of the dying cells^22^. This leads to weakened macrophage recruitment and decreased autophagy level. Evidence suggests that prolonged, excessive, or impaired inflammatory response after MI show associations with increased dilation, expanded infarct and aggravated fibrosis, and might worsen heart dysfunction^18^. We found that the chronic heart failure model was under a low-grade inflammatory state, had a positive correlation with cardiomyocyte apoptosis, inhibited autophagic reactions, and showed a continuous deterioration of cardiac function. However, treatment with QD and valsartan, respectively, resulted in alleviated chronic myocyte inflammation, increased autophagy level, reduced cardiomyocyte apoptosis, alleviated ventricular remodeling, and preserved cardiac function. This was particularly prominent in the QD-M group. These results indicate that QD improves chronic inflammation in CHF and reduces inflammatory-related myocardial apoptosis. Additionally, autophagy upregulation may be an effective mechanism for alleviating further myocardial injury and cardiac insufficiency after MI.

Mechanistic target of rapamycin (mTOR) represents a critical regulatory protein involved in protein production, cell growth/proliferation, autophagy, lysosomal function and metabolic pathways, and is considered a central regulator of cell growth^23^. It is admitted that mTOR is a primary suppressor of autophagy, which acts by controlling essential effectors at the transcriptional and post-transcriptional levels, and by directly phosphorylating the autophagy machinery^24^. For instance, it inhibits autophagy at transcriptional and post-transcriptional levels, and suppresses the formation of autophagosomes and autophagic lysosomes, thereby obstructing the activity of focal adhesion kinase family interacting protein of 200 (ULK1-Atg13-FIP200), which is an essential factor in autophagy occurrence^13^. A study by Buss^25^ found that autophagy induction by inhibiting mTOR could alleviate ventricular remodeling after MI. In addition, mTOR and a p70S6k inhibitor were employed to decrease myocardial cell apoptosis and alleviate ischemic injury after MI^26^. These findings suggest that mTOR suppression and autophagy induction might be important for the protection of cardiomyocytes. We found that mTOR levels were inversely proportional to LC3 amounts in rats with CHF after MI. However, QD could reverse this effect, promote autophagy, and protect heart function.

Eukaryotic translation initiation factor 4E binding protein I (4E-BP1) and 70-kDa ribosomal protein kinase I (p70) induce cell proliferation downstream of mTOR. We found that the trend of p70S6k expression basically followed that of mTOR, but 4E-BP1 amounts were unaltered. Therefore, we hypothesized that mTOR/p70S6k signaling mediates the effects of QD in CHF by inducing autophagy. MI can lead to adverse remodeling in the non-infarcted myocardium^2^, and substantial alterations of cardiac energy metabolism have been detected during remodeling, contributing to CHF severity^27^. Karwi and collaborators^28^ found that inducing catabolic pathways degrading branched chain amino acids reduce heart dysfunction and deleterious remodeling after MI, in association with mTOR/p70S6k signaling suppression. QD may reduce cardiomyocyte apoptosis through the mTOR/p70S6k pathway, which also has a positive inotropic effect to maintain cardiac energy metabolism. This may be the so-called Tonifying Qi in traditional Chinese medicine, but additional investigation is warranted to elucidate the mechanisms by which QD regulates myocardial energy metabolism.

In summary, this study demonstrated that QD exerts its effects in CHF following MI by inhibiting sustained injury resulting from inflammation, reducing myocardial apoptosis, and creating a good microenvironment for healthy myocardium. Further mechanistic investigation revealed that QD may activate autophagy in cardiac myocytes via mTOR/p70S6k pathway suppression. The present findings highlight a new regulatory function for QD in mTOR/p70S6K signaling in CHF, further validating the use of this traditional Chinese medicine formulation for CHF treatment.

## Materials and methods

### **Animal**s

Ninety male Sprague-Dawley (SD) rats (220–240 g) from Beijing Vital River Laboratory Animal Technology (Certificate no.: SCXK (Jing) 2012–0001) were housed under a 12h-12h dark/light cycle at 21 ± 2 °C and 30–70% relative humidity, with freely available rodent chow and water. Experiments assessing animals had approval from Tianjin University of Chinese Medicine (TCM-LAEC20170049), and all experiments complied with the Guide for the Institutional Animal Care and Use Committee (IACUC).

### Drugs and reagents

Qi Dan Li Xin Pill (QD) was provided by the First Teaching Hospital of Tianjin University of TCM (1 g medicinal powder from approximately 3.45 g dried medicinal herbs). Valsartan (Batch number: X2062) was provided by Beijing Novartis Pharma (China). Qi Dan Li Xin Pill (QD) was administered at 2.2 g/kg·d. Valsartan in saline was prepared at 1 mg/mL, and 10 mL/kg was administered.

### Animal model of CHF and treatments

MI induction in the animals was performed by total LADCA ligation upon anesthesia based on recently published protocols^2^. Anesthesia was carried out by intraperitoneally injecting 5% chloral hydrate (300 mg/kg). Throughout the study, 20–25% of rats died, mostly during or following coronary ligation surgery, likely due to acute heart failure or cardiac arrest.

Echocardiographic assessments were performed at 28 days after surgery, and rats were randomized into six groups (left ventricular ejection fraction [EF] between 38% and 50%) based on echocardiographic data and treated immediately. The sham and coronary ligature groups were administered normal saline intragastrically; the remaining groups included the valsartan (Val, 10 mg/kg·d), QD Low dose (QD-L, 1.1 g/kg·d), QD Medium dose (QD-M, 2.2 g/kg·d) and QD High dose (QD-H, 4.4 g/kg·d) groups, which were also treated intragastrically. Following a 28-day treatment course, death rates in the sham, coronary ligature, valsartan, QD-L, QD-M and QD-H groups were 0%, 30%, 10%, 0%, 20% and 25%, respectively. The sample sizes for different groups in assessing various parameters are shown in Table 1.

**Table 1:** Real-time PCR primers.

mRNA (rat)		Primer
IL-6	Forward	GCCAGAGTCATTCAGAGCAATA
	Reverse	GTTGGATGGTCTTGGTCCTTAG
IL-1β	Forward	AGTGAGGAGAATGACCTGTTC
	Reverse	CGAGATGCTGCTGTGAGATT
IL-10	Forward	GGCAGTGGAGCAGGTGAAGAATG
	Reverse	TGTCACGTAGGCTTCTATGCAGTTG
TNF-α	Forward	GCAGATGGGCTGTACCTTATC
	Reverse	GGCTGACTTTCTCCTGGTATG
GAPDH	Forward	ATGATTCTACCCACGGCAAG
	Reverse	CTGGAAGATGGTGATGGGTT

### Echocardiographic and hemodynamic assessments of left ventricular function

Echocardiographic measurements were performed 4 times, including the day before surgery, and at 1, 28 and 56 days post-surgery. Long-axis views were obtained on a Vevo 2100 Ultrahigh resolution small animal ultrasound imaging system in real time (Visual Sonics Vevo 2100, Canada) using a 16/20-Hz echocardiographic transducer (MS250, model C5). The indexes evaluated included left ventricular end-systolic (LVESD) and left ventricular end-diastolic (LVEDD) diameters^2^. Then, EF was derived, as a critical index of left ventricular systolic function. The ultrasound system’s software was employed for analysis^29^.

After 28 days of treatment, the animals underwent anesthesia via catheter insertion into the left ventricle through the carotid artery. Hemodynamic indexes, including HR, left ventricular end-diastolic pressure (LVEDP), LVSP, dp/dt max, and −dp/dt max, were assessed at baseline on an MP100-CE biofunction evaluation system (BIOPAC Systems, USA). When the above operations were completed, rats were euthanized by isoflurane overdose, and heart samples were collected for subsequent experiments, including histopathology and proteomics.

### Histopathology

Myocyte size and interstitial fibrosis were determined histopathologically. Heart fixation was performed with 4% formalin, followed by paraffin embedding. To observe the level of fibrosis around the infarcted area, the specimens were sectioned at 4 µm, submitted to hematoxylin and eosin (H&E) staining, and analyzed by microscopy at 400×.

### Quantitative real-time PCR (qRT-PCR)

Total RNA isolation from rat LV (non-infarcted LV myocardium surrounding the infarcted area) specimens with TRIzol (Invitrogen) as directed by the manufacturer. Reverse transcription was carried out with oligo (dT) primers and Superscript II (Roche). Next, the mRNA amounts of cardiac genes were assessed by qRT-PCR on a Bio-Rad CFX system. Table 1 shows the primers and probes **for** IL-6, IL-1β, IL-10, TNF-α and GAPDH amplification.

### Immunoblot

Myocardial tissue samples were obtained from the infarct’s border zone. Total protein extraction was performed, and protein quantitation was was carried out utilizing a Bicinchoninic acid (BCA) protein assay kit. Equal amounts of protein (30 μg) were resolved by 8% SDS-PAGE and underwent transfer onto polyvinylidene difluoride (PVDF) membranes^30^. After incubation with rabbit-derived primary antibodies^31^ targeting caspase (19677–1-AP; Proteintech, China), cleaved caspase3 (9661; Cell Signaling Technology, MA), Bax (50599-2-Ig; Proteintech), Bcl-2 (26593-1-AP; Proteintech), LC3 (14600-1-AP; Proteintech), mTOR (2972; Cell Signaling Technology), phospho-mTOR (5536; Cell Signaling Technology), p70S6k (AF6226; Affinity Biosciences, USA), phospho-p70S6k (Ser371) (9208; Cell Signaling Technology), phospho-p70S6k (Thr389) (9234; Cell Signaling Technology), 4E-BP1 (AF6431; Affinity Biosciences), and phospho-4E-BP1 (2855; Cell Signaling Technology), respectively, the membranes underwent further incubation with secondary antibodies (2 h at ambient). The chemiluminescence HRP substrate (Millipore Corporation, USA) was employed for visualization. A multifunctional imaging analysis system (VersaDoc MP 5000; Bio-Rad, USA) was used for quantitation.

### Statistics

Data analysis was carried out with SPSS 22.0. One-way analysis of variance (ANOVA) was performed for multiple group comparisons. Data are mean ± standard deviation (s.d.). P < 0.05 indicated statistical significance.

## Data Availability

Data available on request from the authors. The data that support the findings of this study are available from the corresponding author, [Guanwei Fan], upon reasonable request.
